# The Tyrosine Kinase Csk Dimerizes through Its SH3 Domain

**DOI:** 10.1371/journal.pone.0007683

**Published:** 2009-11-04

**Authors:** Nicholas M. Levinson, Patrick R. Visperas, John Kuriyan

**Affiliations:** 1 Department of Molecular and Cell Biology, University of California, Berkeley, California, United States of America; 2 Department of Chemistry, University of California, Berkeley, California, United States of America; 3 California Institute for Quantitative Biosciences, University of California, Berkeley, California, United States of America; 4 Howard Hughes Medical Institute, University of California, Berkeley, California, United States of America; 5 Physical Biosciences Division, Lawrence Berkeley National Laboratory, Berkeley, California, United States of America; University of Queensland, Australia

## Abstract

The Src family kinases possess two sites of tyrosine phosphorylation that are critical to the regulation of kinase activity. Autophosphorylation on an activation loop tyrosine residue (Tyr 416 in commonly used chicken c-Src numbering) increases catalytic activity, while phosphorylation of a C-terminal tyrosine (Tyr 527 in c-Src) inhibits activity. The latter modification is achieved by the tyrosine kinase Csk (C-terminal Src Kinase), but the complete inactivation of the Src family kinases also requires the dephosphorylation of the activation loop tyrosine. The SH3 domain of Csk recruits the tyrosine phosphatase PEP, allowing for the coordinated inhibition of Src family kinase activity. We have discovered that Csk forms homodimers through interactions mediated by the SH3 domain in a manner that buries the recognition surface for SH3 ligands. The formation of this dimer would therefore block the recruitment of tyrosine phosphatases and may have important implications for the regulation of Src kinase activity.

## Introduction

Members of the Src family of protein tyrosine kinases, such as c-Src, Lck, Fyn and Hck, are key players in many signalling pathways that regulate cell growth, proliferation and motility [1]. Src kinase activity is tightly controlled through phosphorylation at two regulatory sites, Tyr 416 and Tyr 527 (chicken c-Src numbering) [2]. Autophosphorylation of the Src family kinases on Tyr 416, located within a central regulatory element in the kinase domain known as the activation loop, increases activity by stabilizing the kinase domain in a conformation that promotes catalysis [3]. In contrast, the phosphorylation of Tyr 527 in the C-terminal tail of the Src family kinases by Csk (C-terminal Src Kinase) results in the intramolecular engagement of the tail by the Src Homology 2 (SH2) domain and the concomitant docking of the Src Homology 3 (SH3) domain onto the SH2-kinase linker, which together stabilize the kinase domain in an inactive conformation [4], [5], [6], [7], [8]. Full inactivation of the Src family kinases consequently requires both dephosphorylation of the activation loop and phosphorylation of the C-terminal tail [9].

The kinase domain of Csk is responsible for the recognition of the Src family kinases as its specific substrates, resulting in phosphorylation of the C-terminal tail [10]. The ability of Csk to regulate Src kinase activity in vivo also depends on both the SH2 and SH3 domains of Csk [11], [12]. Unlike the Src family kinases, Csk is not constitutively membrane-localized, and the SH2 domain is required for the recruitment of Csk to the plasma membrane. Several different proteins have been shown to recruit Csk through its SH2 domain, including paxillin [12] and the transmembrane adapter protein Cbp (Csk-binding protein) [13].

The SH3 domain of Csk interacts with several tyrosine phosphatases [14], [15]. In T-cells, Csk associates with the tyrosine phosphatase PEP, which dephosphorylates the activation loop of the Src family kinase Lck [16]. The association of Csk and PEP provides for a coordinated downregulation of Lck through simultaneous phosphorylation of the C-terminus and dephosphorylation of the activation loop. Csk has also been reported to associate with the widely expressed tyrosine phosphatase PTP-PEST in non-haematopoietic cells [15], suggesting that such a coordinated mechanism may be widespread.

Pursuing the observation that Csk is dimeric at high protein concentration (i.e., ∼1 mg/ml or greater) in vitro [17], we have discovered that Csk dimerizes in a manner that is incompatible with the binding of ligands to the SH3 domain. By interfering with the recruitment of tyrosine phosphatases, Csk dimerization could serve to modulate the degree of activation loop phosphorylation and activity of the Src family kinases.

## Results and Discussion

### Dimerization of Csk Requires the SH3 Domain

**Figure 1:**
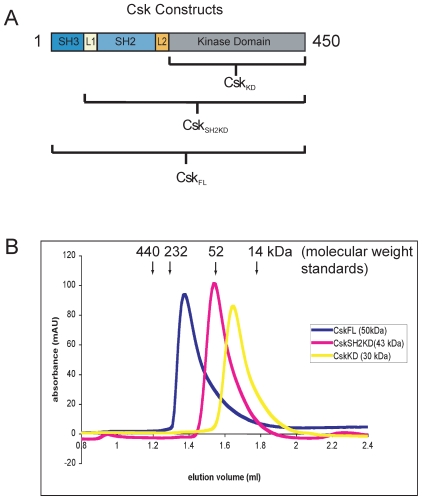
The SH3 domain of Csk is required for dimerization. A) The constructs used in this paper. B) The results of size exclusion chromatography performed with the constructs shown in A. The elution volumes of molecular weight standards are indicated by black arrows.

Csk protein is dimeric in solution, as indicated by analytical ultra-centrifugation and gel filtration chromatography [17], but the molecular nature of this dimer interaction is unknown. We performed size exclusion chromatography with different constructs of Csk (see figure 1), and found that while the full-length protein (Csk_FL_) migrates as a dimer by size exclusion, the removal of the SH3 domain (Csk_SH2KD_) or the SH3 and SH2 domains (Csk_KD_) resulted in proteins that no longer appear to dimerize and instead elute as apparent monomers by gel filtration (figure 1). This suggests that the SH3 domain of Csk is involved in the dimer interface.

### The SH3 Domain Mediates Dimerization of Csk

**Figure 2:**
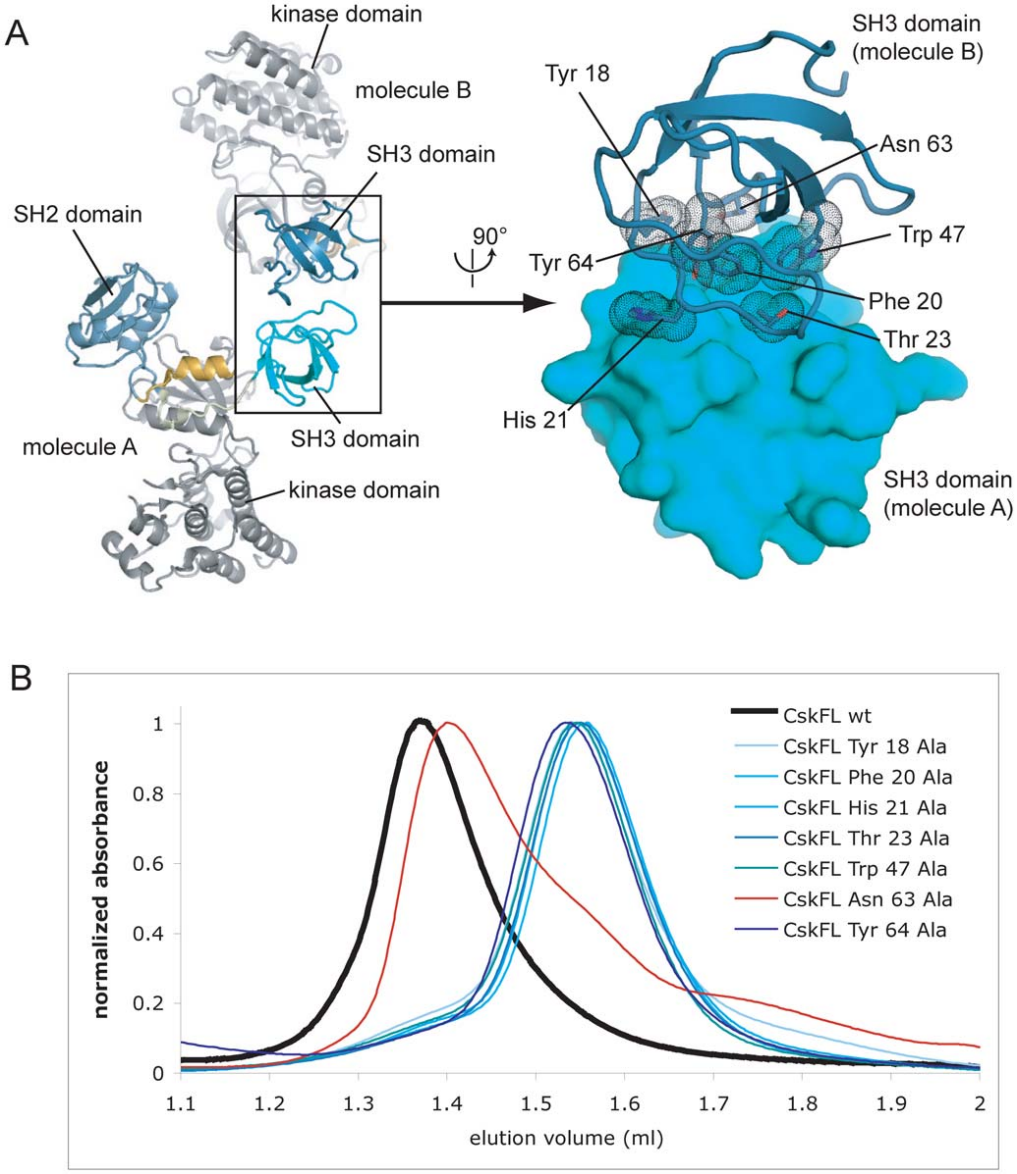
The Csk SH3-SH3 dimer. A) A representative SH3-SH3 dimer from the crystal structure of full-length Csk (PDB code: 1K9A). Residues in the interface are highlighted. B) The results of size exclusion chromatography performed with constructs of Csk bearing mutations in the putative dimer interface.

Analysis of the crystal packing interactions in the crystal structure of full-length Csk [17] reveals an interaction that is consistent with the requirement of the SH3 domain for dimerization. The crystal structure contains 6 molecules of Csk in the asymmetric unit, and pairs of SH3 domains form symmetric interactions consistent with dimer formation. The interaction buries a total of 946 Å^2^ of surface area between the two domains and includes several hydrophobic contacts (figure 2). Despite the fact that the six molecules of Csk in the asymmetric unit of this structure are in somewhat different conformations, the SH3 domains of each molecule participate in identical dimer interactions with adjacent molecules in the crystal lattice. Intriguingly the same interaction is also observed in the crystal structure of the isolated SH3 domain of Csk [18], suggesting that this interaction might be physiologically relevant.

We mutated the surface of the SH3 domain of Csk that mediates the putative dimer interaction extensively, and assessed the ability of the mutant proteins to dimerize by size exclusion chromatography (figure 2). Of the six mutant proteins tested, five migrate as monomers, while the protein bearing the Asn 63 Ala mutation migrates mostly as a dimer, with a small shoulder corresponding to a monomer species. This is consistent with the observation from the structure that Asn 63 makes only very weak interactions in the interface (i.e., it does not form tight hydrogen bonds with neighboring residues). These results indicate that the dimers of Csk observed in solution rely on the SH3–SH3 interface seen in the crystal structures of full-length Csk and the isolated SH3 domain.

### The Csk Dimer Is Incompatible with the Binding of Ligands to the SH3 Domain

SH3 domains bind to peptides containing PXXP sequences that readily form polyproline type II helices [19], [20], [21]. The SH3 domain of Csk binds to a polyproline motif (referred to as 3BP1, for SH3 binding peptide 1) in the C-terminus of the protein tyrosine phosphatase PEP [22], and the recruitment of PEP and Csk to the membrane is required for inhibition of T-cell signaling [16]. The 3BP1 motif of PEP binds to Csk with an unusually high affinity compared to most SH3-peptide interactions, and the high affinity interaction depends on a hydrophobic motif C-terminal to the polyproline motif [22]. The solution structure of the SH3 domain of Csk in complex with a peptide comprising the 3BP1 motif demonstrated that in addition to the canonical polyproline helix-SH3 interaction, the hydrophobic residues in the 3BP1 motif also form interactions with the SH3 domain [23].

**Figure 3:**
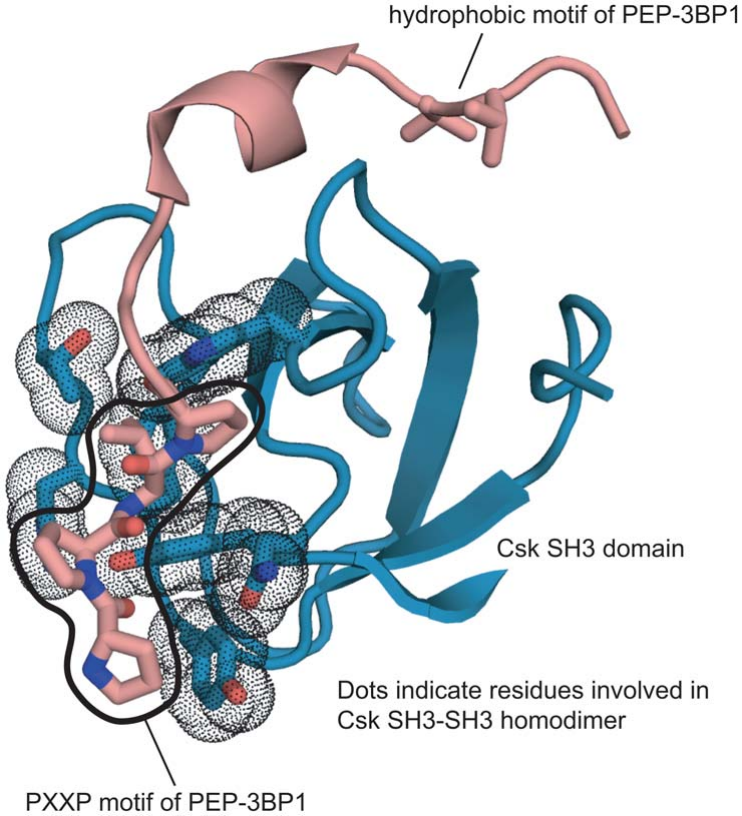
The Csk SH3-SH3 dimer is incompatible with the binding of ligands to the SH3 domain. The solution structure of the SH3 domain of Csk in complex with the phosphatase-derived peptide PEP-3BP1 (pdb code 1JEG). The residues of Csk that are involved in the SH3-SH3 dimer interface are highlighted by sticks and black dots as in Figure 2.

In the Csk SH3-SH3 homodimer the ligand binding surface on both SH3 domains is occluded. This is demonstrated by a comparison between the Csk residues involved in the SH3-SH3 dimer interface with those involved in binding the 3BP1 peptide (figure 3). It is therefore expected that the formation of the SH3-SH3 dimer would prevent the binding of the 3BP1 peptide and vice versa. This is consistent with the observation that the addition of the 3BP1 peptide to the isolated SH3 domain prevents oligomerization of the SH3 domain [23]. Interestingly, the secondary binding site for the hydrophobic residues of 3BP1 is not occluded by the SH3-SH3 dimer (figure 3). It is possible that PEP might still be able to interact weakly with a Csk dimer through this secondary binding site, although the binding of other phosphatases, such as PTP-PEST, to the Csk SH3 domain would be completely blocked by the formation of the dimer.

### A Speculative Model for the Functional Role of the Csk Homodimer

The recruitment of both Csk and PEP to the plasma membrane is critical for the inhibition of T-cell signaling [16]. The observation that the Csk homodimer should interfere with the binding of the PEP phosphatase to the SH3 domain suggests that the two interactions may compete in cells. The dimerization of Csk could therefore have a profound influence on the recruitment of phosphatases to sites of Src kinase activity, with important implications for the regulation of the Src kinases.

The 3BP1 element of PEP interacts with the SH3 domain of Csk with a K_D_ value of ∼800 nM [23]. The affinity of the SH3-SH3 interaction is unknown, but our gel filtration data indicate that the dimer is formed at 20 µM. The question therefore remains whether the SH3-SH3 interaction can effectively compete with the relatively high affinity SH3-PEP interaction. The recruitment of proteins to the cell membrane may increase their effective concentration by as much as 1000-fold [24], allowing interactions that are weak in solution to play a significant role [25]. The phosphorylated cytoplasmic domain of Cbp is known to form oligomers that bind multiple molecules of Csk [26], potentially providing a platform on which Csk molecules are recruited in close proximity. This could further promote the formation of dimers, resulting in the displacement of the phosphatase, and allowing for the activation of Lck through autophosphorylation. In this light it would be interesting to see how mutations that disrupt the Csk SH3-SH3 dimer interface affect signaling in cells.

## Methods

### Expression and Purification of Csk Constructs

The constructs of human Csk, Csk_FL_ (residues 1–450), Csk_SH2KD_ (residues 68–450) and Csk_KD_ (residues 187–450) were expressed in bacteria and purified as described [10]. The SH3 domain mutations were prepared using the Quikchange protocol (Statagene) and verified by DNA sequencing.

### Gel Filtration Chromatography

Purified Csk proteins were subjected to analytical gel filtration using a Superdex S200 column (GE Healthcare) or SMART S200 column (GE Healthcare). Column calibration was performed using molecular weight standards (Ferritin, Catalase, GST, Ribonuclease).
